# Identifying Flow Patterns in a Narrow Channel via Feature Extraction of Conductivity Measurements with a Support Vector Machine

**DOI:** 10.3390/s23041907

**Published:** 2023-02-08

**Authors:** Kai Yang, Jiajia Liu, Min Wang, Hua Wang, Qingtai Xiao

**Affiliations:** 1 State Key Laboratory of Complex Nonferrous Metal Resources Clean Utilization, Kunming University of Science and Technology, Kunming 650093, China; 2 Faculty of Metallurgical and Energy Engineering, Kunming University of Science and Technology, Kunming 650093, China; 3 Department of Management Science and Statistics, The University of Texas at San Antonio, San Antonio, TX 78249-0634, USA

**Keywords:** gas–liquid, flow pattern, rectangular channel, conductivity, support vector machine

## Abstract

In this work, a visualization experiment for rectangular channels was carried out to explore gas–liquid two-phase flow characteristics. Typical flow patterns, including bubble, elastic and mixed flows, were captured by direct imaging technology and the corresponding measurements with fluctuation characteristics were recorded by using an electrical conductivity sensor. Time-domain and frequency-domain characteristics of the corresponding electrical conductivity measurements of each flow pattern were analyzed with a probability density function and a power spectral density curve. The results showed that the feature vectors can be constructed to reflect the time–frequency characteristics of conductivity measurements successfully by introducing the quantized characteristic parameters, including the maximum power of the frequency, the standard deviation of the power spectral density, and the range of the power distribution. Furthermore, the overall recognition rate of the four flow patterns measured by the method was 93.33% based on the support vector machine, and the intelligent two-phase flow-pattern identification method can provide a new technical support for the online recognition of gas–liquid two-phase flow patterns in rectangular channels. It may thus be concluded that this method should be of great significance to ensure the safe and efficient operation of relevant industrial production systems.

## 1. Introduction

Rectangular channels (RCs) with high heat-transfer efficiency and small size have been widely used in small reactors [1], compact heat exchangers [2], and various electronic products [3]. In particular, the gas–liquid two-phase mixing process in rectangular channels involves chemical engineering [4], power engineering [5], etc. Taking the bottom-blow reactor as an example, the mixing state quality of the gas–liquid two phases is closely related to the evolution of the flow patterns. The gas–liquid two-phase flow characteristics are very complicated, due to different gas–liquid mixing modes, different working media, and various intake volumes. Accurate identification of gas–liquid two-phase flow patterns in rectangular channels is not only a basis for obtaining flow parameters, but also directly affects the analysis results of the resistance properties and the heat and mass-transfer properties of the two-phase flow in rectangular channels. In industrial production, some flow patterns not only greatly reduce production efficiency, but even cause serious harm to equipment [6]. Therefore, the investigation of two-phase flow-pattern identification in rectangular channels is of great significance for understanding the heat-transfer mechanism in rectangular channels and ensuring the safe and efficient operation of relevant industrial production systems.

The many approaches to flow-pattern identification can be divided into direct observation and indirect measurement, according to various applicable principles [7]. Direct observation usually adopts a direct convection type, such as a high-speed camera or an X-ray machine, for shooting observation and identification [8,9]. For instance, using a high-speed camera, Schmid et al. obtained a flow pattern map for the adiabatic two-phase flow of carbon dioxide in a vertical upward and downward direction [10]. Cely et al. studied and characterized the gas–liquid slug flow in an annular duct using a high-speed video camera, a wire-mesh sensor, and particle image velocimetry [11]. Skjæraasen et al. studied the measurement of thin liquid films in a gas–liquid pipe flow via X-ray [12]. Azizi et al. investigated bubble column hydrodynamics using ultrafast X-ray computed tomography and radioactive particle tracking [13]. The above studies found that fluid motion can be accurately captured by high-speed cameras, as the information obtained is very rich. However, this depends on the fluid being in transparent containers. In addition, an X-ray machine can be used to scan the gas–liquid mixing process, and the measurements are accurate. However, the identification of flow-pattern has higher requirements on an experimental platform. 

Compared with this direct method, the indirect measurement method is more widely used, especially in the industrial production process, which does not require direct observation of flow patterns in most cases. The indirect measurement method obtains signals such as capacitance [14], pressure [15], temperature [16], and capacitance [17] that can be easily measured, and indicate characteristics of different flow patterns by combining signal-processing technology. Then, the flow pattern or mixing state can be classified and recognized according to these characteristics. For instance, Guo et al. proposed a novel method based on temperature fluctuation for identifying the gas–liquid flow pattern [18]. Oliveira et al. investigated the fluctuations and characteristic frequencies of pressure drop and flow pattern during the flow boiling of isobutane [19]. Perera et al. measured the flow pattern of oil–water mixtures noninvasively by capacitance tomography [20]. The indirect measurement method has a large number of potential engineering applications. However, different industrial scenarios require different measurements. Rectangular channels or, more specifically, bottom-blow reactors, are more difficult to identify because of the impact of application scenarios, sizes, and intake volumes.

With the recent development of computer science, machine learning algorithms have been widely applied with good classification ability [21,22]. In fact, machine learning can be combined with indirect measurement to identify flow patterns quickly. For instance, Zhang et al. studied the identification of oil–gas two-phase flow patterns based on machine learning and electrical capacitance tomography [23]. Sestito et al. classified two-phase flow patterns based on frequency-domain features by machine learning-based classifiers [24]. Amirsoleymani et al. explored two-phase flow-pattern identification in compressed air energy storage systems via dimensional analysis coupled with machine learning [25]. All of the above studies confirmed the reliability of machine learning for flow-pattern recognition. In particular, some machine learning algorithms provide many advantages for small-size samples or nonlinear patterns and, thus, are among the first choices for flow pattern recognition. 

It is worth mentioning that various gas–liquid mixing scenarios can result in various measurements, time series, or signals (see Table 1). With an appropriate method or model, flow patterns can be accurately identified from these signals. Furthermore, the quantized characteristic parameters can be used as the basis for flow pattern judgment. However, the characteristic parameters of different flow patterns still overlap to some extent, and there is some subjectivity and uncertainty in flow pattern recognition. Feature extraction based on measurements, time series, or signals, combined with machine learning to identify flow patterns quickly and accurately, is urgently needed in engineering research. Liang et al. developed a new joint-probability density function of air density and wind speed [26]. Meng et al. analyzed the periodicity and frequency of an interfacial wave by power spectral density (PSD) [27]. Nnabuife et al. attained objective flow-regime identification using spectral features and a support vector machine (SVM) [28]. However, to date, there is no mature theory to describe flow-pattern recognition on the basis of the conductivity measurements in rectangular channels.

Inspired and motivated by all of the above studies, in this work, the issue we needed to address was how to identify and classify the various flow patterns simply and accurately with characteristic parameters of electrical conductivity measurements of the gas–liquid mixing process in rectangular channels. Accordingly, the aim of this work is to propose a two-phase flow-pattern recognition model or framework combining the time-domain and frequency-domain characteristics and a support vector machine. In fact, a unqualified flow pattern not only greatly reduces production efficiency, but also causes serious harm to equipment. This investigation on gas–liquid two-phase flow-pattern identification is of great significance for understanding the heat-transfer mechanism in rectangular channels and ensuring the safe and efficient operation of relevant industrial production systems. The contribution of this work is two-fold. First, using flow data analysis, this article responds to a number of current growing needs for the in-depth mining of sensor data. It provides a systematic solution for the integrative analysis of conductivity measurements, as the electrical conductivity and the flow images were measured and obtained instantaneously. Thus, the subjective experience of operators can be avoided as much as possible for flow-pattern recognition in rectangular channels. Second, this study used a classification methodology to provide a novel and broad framework for combining a feature vector reflecting time–frequency characteristics with a support vector machine. A large number of models and extensions are potential outcomes within this framework, as several signals—such as conductivity, temperature, and pressure in industrial processes—can be input into the algorithms proposed in this work. Hence, the universality of this work can provide a demonstration for various investigators in related fields, including multiphase flow.

The rest of this paper is organized as follows. In Section 2, the gas–liquid two-phase mixing experiment in rectangular channels and the data analysis method for conductivity measurements are described. In Section 3, the main results and discussions of gas–liquid flow-pattern identification in rectangular channels are provided. In Section 4, concluding remarks are presented.

## 2. Materials and Methods

### 2.1. Gas–Liquid Mixing Setup and Materials

**Figure 1:**
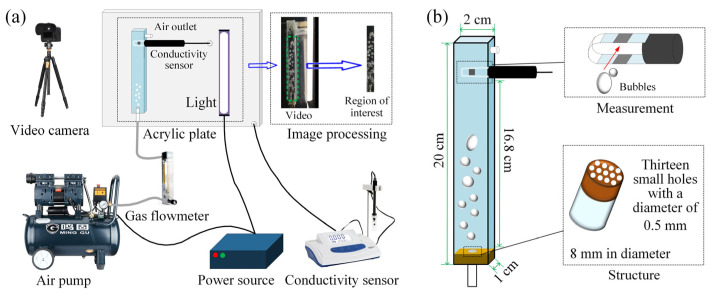
Schematic of the gas–liquid mixing process in a vertical rectangular channel (**a**) and structure and size of the vertical rectangular channel (**b**).

The schematic of the gas–liquid mixing process in a vertical rectangular channel is illustrated in Figure 1a. As shown in this figure, the gas–liquid mixing system consists of an oil-free air compressor (4 × 1500W-20L, Outstanding, Taizhou, China), two intake tubes (8 mm inner diameter, SenDa, Taizhou, China), a glass rotameter (50–500 mL/min, Shuanghuan, Nanjing, China), a rectangular channel (self-made in the laboratory, Kunming, China), a nozzle (self-made in the laboratory, Kunming, China), a conductivity meter (DDSJ-307A, Leici, Shanghai, China), a video camera (equipped with a polarizer, HXR-NX200NX100, Sony, Japan), and a light (L/LT-JY20W, LED, Suzhou, China). The above devices were connected in turn. It is worth mentioning that the conductivity meter was located in the upper part of the rectangular channel. As shown in Figure 1b, the internal width, internal thickness, and considered length of the rectangular channel with the width of 2 cm, thickness of 1 cm, and length of 20 cm were, respectively, 1.6 cm, 0.8 cm, and 16.8 cm. The outside and inside diameters of the nozzle (i.e., the glass tube with the copper sheet covered on the top) fixed at the bottom of rectangular channel were 0.8 cm and 0.6 cm, respectively. Thirteen small holes with a diameter of 0.05 cm were on the top of the nozzle to create more bubbles.

During the experimental procedure, normal-temperature air with density ranging from 1.21 kg/m^3^ to 1.27 kg/m^3^ was used as the gas phase. An NaCl solution with different concentrations was used for the liquid phase. Thirty sets of experiments were designed in order to obtain different flow patterns, as shown in Table 2. The flow rates of air ranged from 50 mL/min to 500 mL/min. At the same time, the rectangular channel was filmed by video camera and instantaneous images of the gas–liquid mixture are obtained via video separation. The gas–liquid mixing process was measured by the conductivity sensor. The measured conductivity varied with the working medium flowing through the conductivity sensor. The conductivity was larger with the NaCl solution inside the sensor, the conductivity was small with bubbles inside the sensor, and the conductivity was between the maximum and minimum levels with the NaCl solution and bubbles inside the sensor.

It is worth mentioning that electrical conductivity varies with the changing of various flow patterns. In fact, the surface tension and viscosity of fluids can indeed affect the evolution of flow patterns, but may not have a remarkable effect on the measurement of electrical conductivity and the identification of flow patterns. In addition, changing the original measurements of electrical conductivity is so very complicated and irregular that it is difficult to understand the flow pattern accurately according to the original direct measurements. However, the abnormal information (i.e., irregular randomness or experimental uncertainty) of the original measurements could be reduced by extracting the features of conductivity measurements to identify the flow patterns in the rectangular channel.

### 2.2. Probability Density Function

The probability density function has often been used to describe the expected values of random variables from a sample in time–frequency domain analysis [33]. According to the conductivity-measurement principle, the conductivity measurements of the gas–liquid two-phase flow in a rectangular channel has the characteristics of time-domain and frequency-domain. Hence, the probability density function can be used to explore the time threshold characteristics of the conductivity measurements. For the conductivity measurements of two-phase flow, its probability density function can reflect the distributional range and the intensity of fluctuation of the measurements, which are closely related to the flow pattern. Therefore, two characteristic parameters, including the standard deviation σ and the skewness $S_{k}$, were introduced to quantify the time-domain characteristics of the typical flow-conductivity measurements from the probability density function point of view. Here, the standard deviation σ reflected the dispersion degree of the conductivity measurements and is calculated as follows:

(1) $$\sigma =\sqrt{\frac{1}{n}\overset{n}{\underset{i=1}{\sum }}{\left(x_{i}-\bar{x}\right)}^{2}}$$

where $x_{i}$ refers to the original conductivity measurement, n refers to the quantity of conductivity measurements, and $\bar{x}$ refers to the average of the original conductivity measurements during the mixing process. The skewness $S_{k}$ is closely related to the shape of the probability density function and is calculated as follows:

(2) $$S_{k}=\frac{\mu_{3}}{\sigma^{3}}=\frac{1}{\sigma^{3}n}\overset{n}{\underset{i=1}{\sum }}{\left(x_{i}-\bar{x}\right)}^{3}$$

where $\mu_{3}$ refers to the third-order center distance of conductivity measurements. The measurement distribution has a negative deviation if the skewness $S_{k}$ is less than zero, whereas the measurement distribution has a positive deviation if the skewness $S_{k}$ is greater than zero. Generally, a normal distribution (i.e., bell curve) exhibits null skewness, but most measurements from engineering practice and laboratory simulation are not absolutely vibratory-symmetric. Greater positive or lower negative skewness is usually caused by operating conditions or interference factors of flow behavior, while lower positive or greater negative skewness can be regarded as the result of abnormal information of flow behavior to a certain extent. Hence, the degree of asymmetry and direction of conductivity-measurement distribution can be determined by measuring the skewness coefficient.

### 2.3. Power Spectral Density

The power spectral density, which is a measure of the mean square value of a random variable, belongs to a probabilistic statistical method and the power spectral density curve shows the relationship between power spectral density and frequency [34]. According to the conductivity-measurement principle, the conductivity measurements of the gas–liquid two-phase flow in a rectangular channel has frequency-domain characteristics. The difference in the conductivity measurements is inevitable in the frequency-domain while the gas–liquid mixtures of different flow patterns pass through the conductivity sensor. Given a conductivity measurement $x_{i}$ that changes with time, the power spectral density $P\left(f\right)$ of the conductivity measurements with the frequency f is defined as follows:

(3) $$P\left(f\right)={\left|\hat{x}\left(f\right)\right|}^{2}$$

where ${\left|\hat{x}\left(f\right)\right|}^{2}$ refers to the square norm of the Fourier transform of the conductivity measurements $x={\left\{x_{i}\right\}}_{i=1}^{n}$ calculated using the fast Fourier transform. In order to quantitatively extract the frequency-domain features of the conductivity measurements, which is similar to the time-domain feature-extraction process, three feature parameters are introduced to further quantify the frequency-domain features of the measurements and construct the feature vector. The three characteristic parameters are the frequency corresponding to the maximum power $f_{\max}$, the standard deviation $\sigma_{P}$ of the power spectral density, and the range $R_{P}$ of the power distribution. For instance, the standard deviation $\sigma_{P}$ of the power spectral density determined as follows:

(4) $$\sigma_{P}=\sqrt{\frac{1}{n}\overset{n}{\underset{i=1}{\sum }}{\left(P\left(f_{i}\right)-\bar{P}\left(f\right)\right)}^{2}}$$

where $P\left(f_{i}\right)$ refers to the power spectral density of the conductivity measurements at frequency $f_{i}$ and $\bar{P}\left(f\right)$ refers to the average of the power spectral density. For the purpose of employing a similar method, some more specific details of power spectral density are present in Ref. [7].

### 2.4. Support Vector Machine

**Figure 2:**
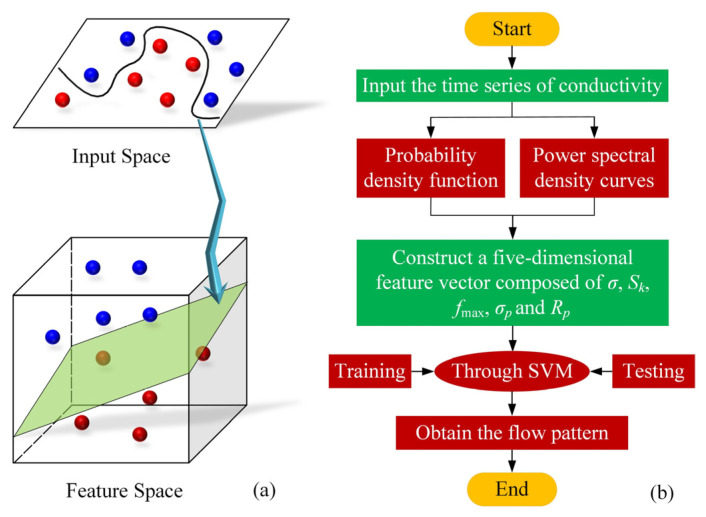
Schematic diagram of support vector machine for classification (**a**) and flowchart of the flow-pattern identification using the conductivity feature-extraction technique combined with support vector machine (**b**).

A support vector machine was originally a binary classification algorithm that supported both linear or nonlinear classifications [35], as shown in Figure 2a. It evolved to support multiple classification problems as well. The learning strategy of a support vector machine is to maximize the interval, which can be formalized as a problem of solving convex quadratic programming and is also equivalent to the regularized hinge loss function minimization problem. In this work, the radial basis function, which is one of the most widely used kernel functions due to its similarity to Gaussian distribution, is considered for support vector machine, and is defined as follows:

(5) $$K\left(X_{i},X_{j}\right)=\exp\left(-\gamma \cdot \|X_{i}\cdot X_{j}\|{}^{2}\right)$$

where $X_{i}$ and $X_{j}$ are input modes and γ is the parameter. The construction of a suitable feature vector is the basis of a support vector machine. Based on the time-domain and frequency-domain characteristic parameters obtained by the above calculation, a five-dimensional eigenvector composed of σ, $S_{k}$, $f_{\max}$, $\sigma_{P}$, and $R_{P}$ was constructed.

In addition, Figure 2b shows a flowchart of flow-pattern identification using conductivity feature extraction combined with a support vector machine. The procedure is as follows: (1) Five-dimensional feature vectors of different gas–liquid flow patterns in a rectangular channel are extracted; (2) Several five-dimensional eigenvectors of different gas–liquid flow patterns in the rectangular channel are sent to the support vector machine; (3) Five-dimensional feature vectors of different gas–liquid flow patterns in the rectangular channel are trained and tested in the support vector machine; (4) The training sets of the five-dimensional feature vectors of the different flow patterns are obtained; (5) A conductivity time series is entered; (6) The probability density function and the power spectral density of the conductivity time series are obtained; (7) A five-dimensional eigenvector composed of σ, $S_{k}$, $f_{\max}$, $\sigma_{P}$, and $R_{P}$ is constructed; (8) The five-dimensional eigenvectors are sent to the support vector machine; (9) The classification results of the flow patterns are obtained. Note that each feature vector contains a label to indicate its corresponding flow pattern. The number 1 indicates the bubble flow, 2 indicates the slug flow, and 3 indicates the mixed flow.

## 3. Results and Discussion

### 3.1. Relationship between Flow Pattern and Conductivity

**Figure 3:**
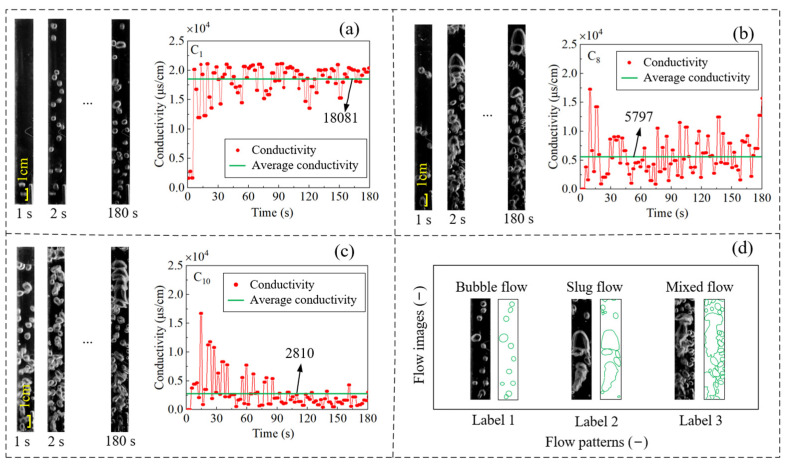
Pictures captured and conductivity measurements of three gas–liquid flow patterns in rectangular channel: bubble flow (**a**), slug flow (**b**), mixed flow (**c**), and three categories of flow pattern (**d**).

The flow pattern images of the gas–liquid mixing process in rectangular channel under a variety of working conditions were recorded. At the same time, the conductivity of the gas–liquid mixing process was recorded by a conductivity sensor. It is worth mentioning that the conductivity of the gas–liquid mixture changes with various flow patterns. In other words, the fluctuant conductivity measurements are recorded as bubbles, gas–liquid mixtures, and liquids flowing through the conductivity sensor. Figure 3 shows three typical flow patterns, including the bubble flow, the slug flow, and the mixed flow, observed experimentally, and the corresponding conductivity measurements.

As shown in Figure 3a, the bubbles are dispersed in a rectangular channel and can flow freely while the gas flow rate is low. In particular, the shape of the bubbles is regular and the bubbles are distributed independently. In this case, the resulting flow pattern was the bubble flow. As shown in Figure 3b, the bubble size became larger and was squeezed and deformed by liquid with the increase in gas velocity and flow rate. Adjacent bubbles gradually began to collide and fuse into long and thin bubbles, and the flow pattern gradually changed from the bubble flow to the slug flow. As can be seen, the bubbles were no longer distributed independently, but were attached to each other to form large, irregularly shaped bubbles. Due to the unique structure of the rectangular channel, the tail of the elastic bubble followed a group of bubble flows during the flow process. The size of the bubble increased gradually with the further increase in gas velocity. The bubble swallowed up other bubbles around it. Then, the slug flow changed into other flow patterns. Figure 3c shows a typical mixed flow. It can be seen that the liquid phase has been unable to form a continuous channel, while the gas phase has formed a continuous channel. The bubbles have connected to each other, forming larger, more irregular bubbles or clusters of bubbles. Since various irregular forms of bubbles exist in the mixed flow, it is difficult to tell what individual bubbles look like. This is also typical of the mixed flow pattern. Figure 3d shows a schematic diagram of the above three flow patterns. The corresponding conductivity measurements of the three typical flow patterns were further analyzed. It was noted that among the conductivity measurements corresponding to the three flow patterns, the amplitude of the mixed flow measurements was the lowest. The bubble size was larger in the elastic flow. As a result, bubbles or groups of bubbles could pass independently through the conductivity sensor in the rectangular channel, resulting in smaller conductivity fluctuations and smaller measurements. However, the conductivity corresponding to the bubble flow was the greatest. Because the bubbles were distributed independently, the gas and liquid pass alternately through the conductivity sensor. The conductivity was small when bubbles passed through, whereas the conductivity was greater when the liquid passed through. In addition, the conductivity amplitude of the elastic flow was large. There were large bubbles in the elastic flow, and large bubbles could take up all the space of the conductivity sensor. This resulted in small conductivity measurements. However, after the slug bubble flowed through the conductivity sensor, the liquid did. This resulted in bigger conductivity.

In fact, the mean of the conductivity measurements of different flow patterns was also different. It can be observed from Figure 3 that the average conductivity of the bubble flow was 18,081 μs/cm, the average conductivity of the slug flow was 5797 μs/cm, and the mean of the mixed flow was 2810 μs/cm. There was little difference between the average conductivity of the slug flow and that of the mixed flow, whereas the average conductivity of the bubble flow was very large and was different from that of the slug flow and the mixed flow. This was mainly because the bubbles of the bubble flow were independently distributed and the movement speed was small, so the conductivity sensor recorded more conductivity of liquid. However, it was difficult to guarantee the accuracy of distinguishing the flow patterns in the rectangular channel by observation, due to the lack of accurate evaluation indices and rapid classification methods. Therefore, the characteristic parameters of the conductivity measurements needed to be extracted. The characteristic parameters could be used to describe flow patterns. By feeding these characteristic parameters into the support vector machine, machine learning and flow classification could be achieved.

### 3.2. Time-Domain Feature Extraction of Conductivity 

**Figure 4:**
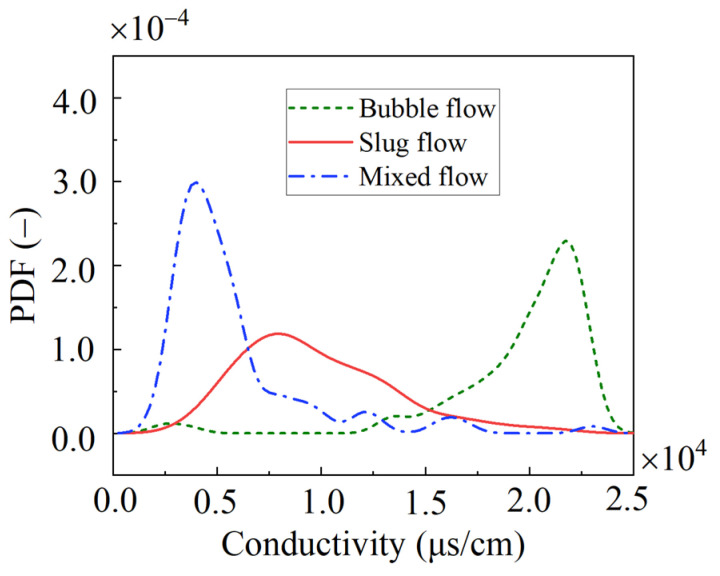
Probability density function curves of conductivity measurements corresponding to the three flow patterns in the vertical rectangular channel.

In order to extract the time-domain features of the conductivity time series, the probability density function was first used to analyze the conductivity measurements in a vertical rectangular channel. The probability density function curves of the conductivity measurements of different flow patterns were obtained via calculation software and are shown in Figure 4. It can be seen that although these probability density function curves were unimodal curves, the probability density function curves of the mixed flow had the highest peak value and the most concentrated conductivity distribution, due to the collision and deformation of bubbles at this time. However, due to the large intake volume, the conductivity fluctuation was also small. The probability density function curve of the slug flow had the smallest peak value and the widest distribution range of conductivity, which indicated that the slug flow measurements fluctuated the most violently among the three flow patterns. At this moment, as the gas velocity increased to a certain extent, the gas–liquid phase ratio in the flow passage was close, and both of them moved violently, alternately, in the flow passage. In the meantime, this violent movement was further reflected in the conductivity measurement probability density function curve characteristics. Moreover, although its gas velocity of the bubble flow was lower than that of the elastic flow, the bubbles were all independently distributed in the rectangular channel. The liquid phase formed a continuous phase, occupying the main space within the rectangular channel. At the same time, the liquid was distributed around all the intact bubbles and was moved together by the gas phase. The intensity of the interaction between the two phases and between the bubbles decreased in the rectangular channel, so the conductivity fluctuation was lower than that of the slug flow, but it was still higher than that of the mixed flow.

**Figure 5:**
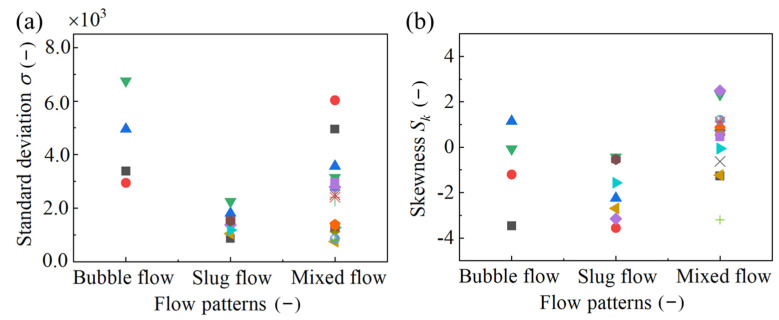
Distribution of standard deviation and skewness of conductivity measurements for the three flow patterns in a rectangular channel: standard deviations (**a**) and skewness (**b**). (Symbols with individual type and color used here refer to various working conditions.).

In order to further quantify the time-domain characteristics of the typical flow conductivity measurements, the standard deviation σ and the skewness $S_{k}$ of the distribution were introduced. After processing 30 groups of typical flow patterns, the ranges of distributional parameter of the three flow patterns were obtained, as shown in Figure 5. It is worth mentioning that in order to facilitate the later training and testing of machine learning, the 30 sets of data were divided into two groups, according to time. The first group, which contained 30 sets of conductivity measurements with times of 0~90 s, was used for feature parameter extraction and training. The second group, which also contained 30 sets of conductivity measurements with times of 91~180 s, was used to test the accuracy of flow-pattern recognition. As can be seen from Figure 5a, the dispersion degree of its conductivity-measurement distribution was low, while the standard deviation of the slug flow was small. The standard deviation of the bubble flow was significantly higher than that of the other two flow patterns, indicating that the conductivity-measurement distribution of the bubble flow was highly discrete. Moreover, it was consistent with the previous conclusions based on the probability density function curve. Meanwhile, it can be observed from Figure 5a that the standard deviation distribution range of the bubble flow and the mixed flow was wider. In particular, the standard deviation of the mixed flow was between 7.59 × 10^2^ and 6.03 × 10^3^, indicating that mixed flow was an unstable flow pattern. In general, the difference between the standard deviations of these three flow patterns was relatively large. Hence, the standard deviation σ of the conductivity measurements could be used as the characteristic parameter of the flow pattern. More importantly, it can be noted from Figure 5b that the skewness $S_{k}$ of the slug flow was less than zero, showing that the measurements distribution was negative skew. However, for the bubble flow and the mixed flow, the skewness $S_{k}$ was both less than and greater than zero. The measurement distribution was more complex than it was for the other two flow patterns. 

The specific distribution of the standard deviation and the skewness of the three flow patterns in the rectangular channel are shown in Table 3. It can be observed from the table that the measurements of the elastic flow were indeed negative skew. The skewness $S_{k}$ of the bubble flow was distributed from −3.47 to 1.14, the skewness $S_{k}$ of the elastic flow was distributed from $-5.40\times 10^{-1}$ to −1.57, and the skewness $S_{k}$ of the mixed flow was distributed from −5.86 × 10^−2^ to 2.49. Obviously, the skewness $S_{k}$ of the three flow patterns was different, but the distribution width of skewness was roughly equal. It can be concluded that skewness $S_{k}$ can be used as a characteristic parameter for the flow-pattern recognition of the gas–liquid mixing process in the rectangular channel.

### 3.3. Frequency-Domain Feature Extraction of Conductivity

**Figure 6:**
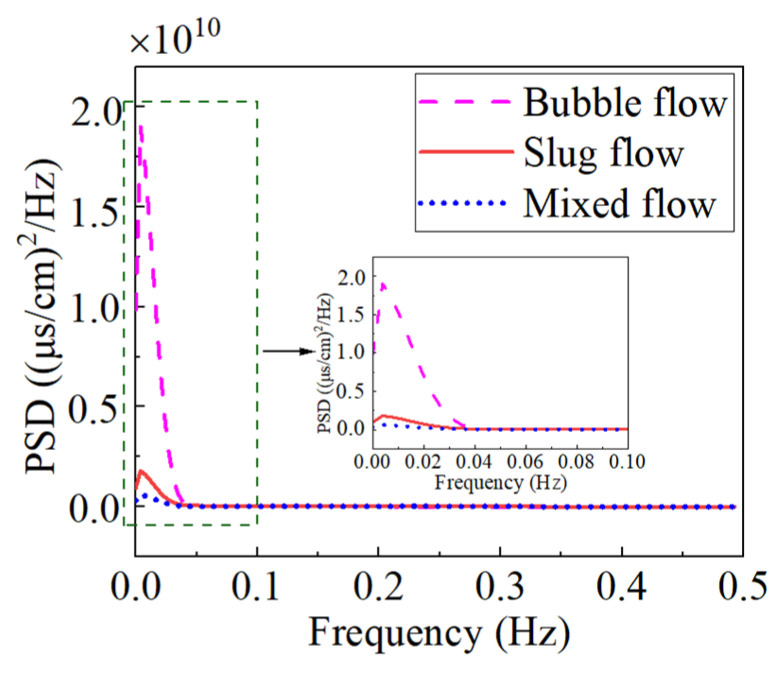
Power spectral density distribution of conductivity measurements corresponding to the three flow patterns in the vertical rectangular channel.

On the basis of time-domain analysis, the conductivity measurements were processed by the power spectral density with frequency-domain analysis. The power spectral density curves of the three typical flow patterns were obtained and are shown in Figure 6. It may be observed that the power spectral density curves of the three flow patterns had only one peak. What is more interesting is that the peaks of all three power spectral density curves were located near 3.85 × 10^−3^ Hz, showing that the power distribution of the three flow patterns was very concentrated. Meanwhile, it can also be seen that the peak of the bubble flow was the largest. The peaks of the elastic flow and the mixed flow were relatively small, and the difference between them was not big. Compared with those of the bubble flow and the slug flow, the power distribution of the mixed flow behaved more uniformly. The difference in peaks did not suggest different flow patterns. However, other characteristic parameters need to be introduced to assist when the peaks are close. 

**Figure 7:**
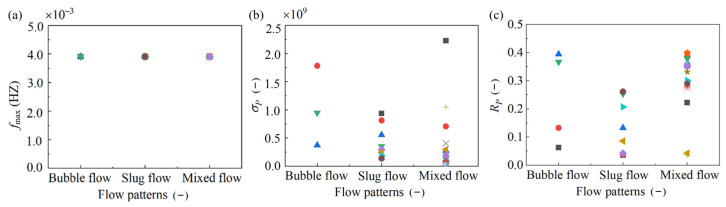
Parameter distribution of three flow patterns of power spectral density: frequency $f_{\max}$ corresponding to the maximum power (**a**), the standard deviation $\sigma_{P}$ of the power spectral density (**b**), and the range $R_{P}$ of power distribution (**c**). (Symbols with individual type and color used here refer to various working conditions.).

In order to further quantitatively extract frequency-domain features of conductivity measurements, three characteristic parameters—frequency corresponding to the maximum power $f_{\max}$, the standard deviation $\sigma_{P}$ of the power spectral density, and the power distribution range $R_{P}$—were introduced. The distribution ranges of the three characteristic parameters of the three flow patterns are shown in Figure 7. It is worth mentioning that in this work 99% of the total power was distributed between 0 and $R_{P}$. As can be seen from Figure 7a, $f_{\max}$ of the three flow types, including the bubbly flow, the elastic flow, and the mixed flow, were equal. This meant that $f_{\max}$ could not be used to describe the flow characteristics, whereas $f_{\max}$ was universal in describing flow characteristics based on signal processing. Hence, to provide a reference for other researchers, $f_{\max}$ was retained in this work. The standard deviation $\sigma_{P}$ of the power spectral density reflected the dispersion degree of the power distribution of the three flow patterns. As can be seen from Figure 7b, the values of $\sigma_{P}$ of the slug flow and the mixed flow were small, while that of the bubble flow was large. Meanwhile, the values of $\sigma_{P}$ of the power spectral density of the slug flow and the mixed flow were distributed in a smaller width, while that of the bubble flow was distributed in a larger width, which was consistent with the peak distribution characteristics of the three flow patterns. This showed that the power distributions of the slug flow and the mixed flow were less discrete, while that of the bubble flow was more discrete. Therefore, $\sigma_{P}$ could be used as a parameter to describe the characteristics of the gas–liquid two-phase flow patterns in the rectangular channel. Parameter $R_{P}$ refers to the power distribution range of the conductivity measurements. Figure 7c shows the total power distribution of the three flow patterns. It is noted that the power distribution range of the mixed flow was large and $R_{P}$ was relatively concentrated. The distribution of $R_{P}$ in the slug flow was relatively uniform. The distribution of $R_{P}$ in the bubble flow was relatively dispersed. In general, the total power distribution of the three flow modes was quite different.

Moreover, the ranges of distribution parameters of the power spectral density of the conductivity measurements in the rectangular channel are shown in Table 4. The data in this table visually show the distribution range of each parameter. The distribution ranges $R_{P}$ of the bubble flow and the mixed flow were similar. However, $R_{P}$ of the bubble flow was more dispersed. This indicated that the distribution of $R_{P}$ in the mixed flow was more obvious than that of the bubble flow. At the same time, the distribution of $R_{P}$ in the slug flow was also more obvious than that in the bubble flow. Therefore, $R_{P}$ could be used to describe the flow-pattern characteristic in the rectangular channel. In general, the maximum power $f_{\max}$, the standard deviation $\sigma_{P}$ of the power spectral density, and the power distribution range $R_{P}$ were significantly different under the different flow patterns. All three parameters could be used to describe the flow patterns. In particular, in this work, $\sigma_{P}$ and $R_{P}$ were mainly used to describe the gas–liquid flow pattern characteristics.

### 3.4. Flow Pattern Recognition Based on SVM

The time-domain and frequency-domain characteristics of the conductivity measurements of three typical flow patterns during gas–liquid two-phase mixing in rectangular channel were analyzed and quantified. It can be seen that the five quantized characteristic parameters—σ, $S_{k}$, the frequency corresponding to $f_{\max}$, $\sigma_{P}$, and $R_{P}$—can be used as the basis for the judgment of flow pattern. However, these characteristic parameters of different flow patterns still have some crossover, resulting in some subjectivity and uncertainty in flow-pattern recognition. Therefore, based on the above research results, the support vector machine was further constructed in this work to obtain more rapid, efficient, and accurate flow-pattern-classification results.

First, several five-dimensional feature vectors consisting of σ, $S_{k}$, $f_{\max}$, $\sigma_{P}$ and $R_{P}$ were constructed. These vectors could be used as indicators to identify the flow pattern of the gas–liquid mixing process in the rectangular channel. In addition, each feature vector contained a label to indicate its corresponding flow patterns. Label 1 referred to the bubble flow, label 2 referred to the slug flow, and label 3 referred to the mixed flow. Table 5 shows some feature vectors for different flow patterns. In this table, the characteristics of the three flow patterns are described quantitatively. In the case of the bubble flow, they were as follows: $\sigma =3.39\times 10^{3}$, $S_{k}=-3.47$, $f_{\max}=3.91\times 10^{-3}$, $\sigma_{P}=2.58\times 10^{9}$, and $R_{P}=6.25\times 10^{-2}$. All feature vectors were sent to the support vector machine for learning and training. It is worth mentioning that a total of 30 sets of data were obtained in this experiment, including four sets of bubble flow data, 18 sets of elastic flow data, and eight sets of mixed flow data. The pre-90 s of data in each set was used for training, and the other 90 s was used for testing. The data number of the bubble flow, the slug flow and the mixed flow in the training set was 4, 18, and 8, respectively. The data number of the bubble flow, the slug flow, and the mixed flow in the test set was 4, 18, and 8, respectively. 

Table 6 shows the test set identification results of the three flow patterns for the gas–liquid mixing process in the rectangular channel. In this table, it can be seen that the model correctly identifies all bubble flows. However, one set of data in the slug flow was identified as the bubble flow. One set of data in the mixed flow was identified as the slug flow. The overall recognition accuracy was 93.33%. In general, it was feasible to analyze the time-domain and frequency-domain characteristics of the gas–liquid two-phase conductivity measurements in the rectangular channel and to construct the feature vector reflecting the time–frequency characteristics of the conductivity measurements. The learning and training of the feature vectors describing the flow patterns was conducive to the construction of the support vector machine model. The established machine learning model could quickly and efficiently identify and classify the flow patterns of the gas–liquid mixing process in the rectangular channel.

## 4. Conclusions

In this work, the gas–liquid mixing process in the rectangular channel was measured, and the flow pattern images and conductivity measurements of the bubble flow, the slug flow, and the mixed flow were obtained. The time-domain and frequency-domain characteristics of the conductivity measurements were analyzed by the probability density function and power spectral density. The feature vector describing the flow pattern in the rectangular channel was constructed by introducing characteristic parameters. The classification and recognition of gas–liquid two-phase flow patterns in the rectangular channel was realized by using a support vector machine, and good results were obtained. The main conclusions can be summarized as follows: (1) The relationship between the gas–liquid flow patterns and electrical conductivity in the rectangular channel was discussed in detail. The bubble flow, the slug flow, and the mixed flow were measured in different conductivity while passing through the conductivity sensor. The evolution of the flow pattern could be qualitatively analyzed by observing the fluctuation of the conductivity measurements. (2) The probability density function and power spectral density were used to process the conductivity measurements of the gas–liquid two-phase mixing process in the rectangular channel for the first time. The five-dimensional feature vector describing the flow pattern was constructed by introducing feature parameters, including σ, $S_{k}$, $f_{\max}$, $\sigma_{P}$, and $R_{P}$. (3) Two time-domain feature parameters and three frequency-domain feature parameters were used as the feature vectors of the flow patterns for training and identifying by the support vector machine. The recognition accuracy of the bubble flow was 100%, and the overall recognition accuracy was 93.33%, showing that the recognition accuracy of this model is reliable. (4) The proposed flow-pattern-recognition framework, combining conductivity measurements and the support vector machine, has the advantages of simplicity, accuracy, and universality for rectangular channels. In fact, the generality of the model and the approach would be extended via extracting the other essential features of different flow patterns in rectangular channels by capturing other signals, including pressure, temperature, and resistance.

## Figures and Tables

**Table 1:** Summary of signal measurement and flow-pattern recognition in gas–liquid mixing process.

Study	Method	Aims	Finding
Chu et al. [6]	Pressure signal	Identification of boiling flow pattern	More than 75% of spectrograms of rolling conditions could be identified.
Ju et al. [29]	Digital imaging	Flow image segmentation and bubble-pattern extraction	Individual small and large bubbles in a direct-contact mixing system could be identified.
Liu et al. [30]	Pressure signal	Recognition of gas–liquid two-phase flow patterns	Both optimal single and combined signals for flow patterns recognition could be obtained.
Liu et al. [31]	Machine learning	Two-phase flow-pattern identification	RUSBoost tree performed best with an accuracy of 97.4%.
Li et al. [32]	wavelet multiresolution	Identification of two-phase flow patterns	Average recognition rate of three flow patterns was higher than 94.2% under certain condition.
Liang et al. [33]	Ultrasonic echoes	Identification of gas–liquid flow patterns in a horizontal pipe	Stratified flow, slug flow, and annular flow were identified with an accuracy of 94.0%.
Wu et al. [34]	Special forecasting rules	Qualitative prediction of the formation position of the liquid slug	All the cases of the transition to undesirable flow patterns were successfully forecasted.
Xu et al. [35]	Pressure difference	Feature extraction and selection	The influence of the measurement distance and location on the recognition rate was revealed.

**Table 2:** Experimental design of gas–liquid mixing in the rectangular channel.

	Air Intake	Unit: mL/min									
NaCl Solution		50	100	150	200	250	300	350	400	450	500
Percentage	1.2%	C_1_	C_2_	C_3_	C_4_	C_5_	C_6_	C_7_	C_8_	C_9_	C_10_
	0.75%	C_11_	C_12_	C_13_	C_14_	C_15_	C_16_	C_17_	C_18_	C_19_	C_20_
	0.25%	C_21_	C_22_	C_23_	C_24_	C_25_	C_26_	C_27_	C_28_	C_29_	C_30_

**Table 3:** Specific distribution of standard deviation and skewness of three flow patterns in the rectangular channel.

Flow Patterns	Range	
	σ	*S_k_*
Bubble flow	2.95 × 10^3^~6.75 × 10^3^	−3.47~1.14
Slug flow	1.06 × 10^3^~8.70 × 10^3^	−5.40 × 10^−1^~−1.57
Mixed flow	7.59 × 10^2^~6.03 × 10^3^	−5.86 × 10^−2^~2.49

**Table 4:** Parameter distribution range of power spectral density of conductivity measurements in the rectangular channel.

Flow Patterns	Range		
	$f_{max}$	$\sigma_{P}$	$R_{P}$
Bubble flow	$3.91\;\times \;10^{-3}$	$3.72\;\times \;10^{8}$ $\sim 2.58\;\times \;10^{9}$	$6.25\;\times \;10^{-2}$ $\sim 1.33\;\times \;10^{-1}$
Slug flow	$3.91\;\times \;10^{-3}$	$1.36\;\times \;10^{8}$ $\sim 9.36\;\times \;10^{8}$	$8.59\;\times \;10^{-2}$ $\sim 1.33\;\times \;10^{-1}$
Mixed flow	$3.91\;\times \;10^{-3}$	$2.08\;\times \;10^{7}$ $\sim 2.23\;\times \;10^{9}$	$4.30\;\times \;10^{-2}$ $\sim 2.23\;\times \;10^{-1}$

**Table 5:** Examples of five-dimensional feature vectors for flow pattern in the rectangular channel.

σ	$S_{k}$	$f_{max}$	$\sigma_{P}$	$R_{P}$	Label	Flow Patterns
$3.39\;\times \;10^{3}$	−3.47	$3.91\;\times \;10^{-3}$	$2.58\;\times \;10^{9}$	$6.25\;\times \;10^{-2}$	1	Bubble flow
$8.70\;\times \;10^{2}$	−0.51	$3.91\;\times \;10^{-3}$	$9.36\;\times \;10^{8}$	$3.52\;\times \;10^{-2}$	2	Slug flow
$4.95\;\times \;10^{3}$	−1.27	$3.91\;\times \;10^{-3}$	$2.23\;\times \;10^{9}$	$2.23\;\times \;10^{-2}$	3	Mixed flow

**Table 6:** Testing results for flow-pattern identification of gas–liquid mixing in the rectangular channel.

Flow Patterns	Conditions	Number of Test Sets	Correct Identification Number	Accuracy Rate (%)
Bubble flow	C_1_, C_2_, C_3_, C_5_	4	4	100
Slug flow	C_4_, C_6_~C_10_, C_15_~C_19_, C_25_~C_30_	18	17	94.44
Mixed flow	C_11_~C_14_, C_21_~C_24_	8	7	87.5
Total	/	30	28	93.33

## Data Availability

Not applicable.
